# Different Roles of Telehealth and Telemedicine on Medical Tourism: An Empirical Study from Azerbaijan

**DOI:** 10.3390/healthcare9081073

**Published:** 2021-08-20

**Authors:** Dongxiao Gu, Gunay Humbatova, Yi Xie, Xuejie Yang, Oleg Zolotarev, Gongrang Zhang

**Affiliations:** 1 The School of Management, Hefei University of Technology, Hefei 230009, China; mikehfut@gmail.com (D.G.); gunayhumbetova1@gmail.com (G.H.); yixie928@163.com (Y.X.); wangzhaojun97@126.com (G.Z.); 2 Key Laboratory of Process Optimization and Intelligent Decision-Making of Ministry of Education, Hefei 230009, China; 3 The Department of Information Systems in Economics and Management, Russian New University, 105005 Moscow, Russia; ol-zolot@yandex.ru

**Keywords:** medical tourism, user satisfaction, healthcare telecommunication system, internet healthcare services, medical travel intentions

## Abstract

With the rapid progress in mobile healthcare and Internet medicine, the impact of telehealth and telemedicine on the satisfaction of patients and their willingness to travel has become a focus of the academic research community. This study analyses the differences between telehealth and telemedicine and their role in medical tourism. We examine how the information quality and communication quality of telehealth and telemedicine influence patient satisfaction, and their effects on patients’ willingness to undertake medical travel and on their medical travel behaviours. We conducted an empirical study on the use of telehealth and telemedicine and on medical travel behaviour in Azerbaijan using a survey for data collection. A total of 500 results were collected and analysed using SmartPLS 3.0. Results show that (1) the communication quality and information quality of telehealth and telemedicine and their effects on satisfaction have significantly positive influences on willingness to undertake medical travel; (2) the psychological expectations of value and cost (perceived value and perceived cost) have a positive influence on medical travel; and (3) willingness to participate in medical travel positively influences medical travel behaviour. Moreover, results of this study have implications for research on, and the practice of, using telehealth and telemedicine as they relate to medical tourism. This research may help improve knowledge about telehealth and telemedicine and understand the differences between them in detail. This empirical research model may also be useful for researchers from other countries who wish to measure medical travel behaviour.

## 1. Introduction

Compared with the long waiting lists, high treatment costs and lack of travel opportunities associated with medical care at home, the idea of accessing healthcare in another country is more appealing to many people [1]. Medical tourism, where patients travel abroad for treatment, has developed rapidly in recent years. High levels of technology and medical capabilities in the target countries, transportation costs within a budget and positive web marketing play a large role in this development [2]. Medical tourism is a growing dimension of healthcare globalisation, whereby people elect to travel across borders or to overseas countries to receive their treatment [3].

Nowadays, with regard to the development of the Internet and communication technologies, people can easily buy products from all over the world using only Internet network devices and their credit card. The fact that the medical industry now has such opportunities is one of the biggest contributions of science and technology to human life. People can find a senior doctor from anywhere in the world without leaving their homes and have the opportunity to contact them and receive treatment remotely [4].

The World Health Organization, in response to the question, ‘What is telemedicine’? stated that in the 1970s, ‘telemedicine’ was a term coined literally to mean ‘remote recovery’ [5]. Telemedicine could help health professionals obtain medical information about patients through the use of communication technology. Smith [6] gives the following definition to distinguish between telehealth and telemedicine: ‘The term telehealth includes a broad range of technologies and services to provide patient care and improve the healthcare delivery system as a whole. Telehealth and telemedicine are disparate from each other; telehealth expresses a broader content’.

In a new and rapidly changing technological era, the use of technological services has become a common situation even for healthcare services [7]. People use telehealth and telemedicine with information and communication technology in all fields of healthcare. Hence, this study aims to answer following questions:

Can telehealth and telemedicine be distinguished from each other when both are being used?

How does telehealth and telemedicine affect the willingness to participate in medical travel towards medical tourism behaviour?

Previous studies have shown different studies on the types of health technology services on medical tourism [8]. In addition, studies have attempted to show the difference between telemedicine and telehealth [9]. However, only a few studies have examined the role of telehealth and telemedicine in the field of medical tourism. In this study, we further examine the differences between telehealth and telemedicine and then investigate their roles on medical travel behaviour.

The clinical significance of this research will help shed light on what telemedicine and telehealth are and in which aspects they can be helpful for patients. Many articles attempt to explain the meaning and importance of telemedicine and telehealth [10,11], but most of them could not identify much difference between the two. We have reviewed all these articles. Therefore, we believe that we have provided information about telemedicine and telehealth based on our own personal and scientific experiences. The other clinical significance of our research is about the role of telehealth and telemedicine on medical tourism and their influence on medical travel behaviour. To explain these essential pieces of information, we conducted an empirical study on Azerbaijan.

This study contributes to the literature in three aspects. Firstly, it reveals the differences between telehealth and telemedicine in detail (Table 1). Secondly, this study reviews previous studies [12,13,14] and presents a separate explanation of the roles of telehealth [12,15] and telemedicine [16] in health tourism based on the differences we have described. Lastly, this study explains the stage of medical tourism in Azerbaijan [17] and develops a research model on people’s willingness to undertake medical tourism and medical travel behaviours using telehealth and telemedicine. Although telehealth and telemedicine have no detailed explanations yet [9], our distributed questionnaire was successfully completed by respondents. We ensured that our questions were understandable by giving people detailed information appropriate to our research.

The rest of the article is organised as follows: in Section 2 we review the relevant literature. In Section 3, based on the review of relevant theories and research, we develop a research model and propose the hypotheses. Section 4 discusses the research methodology used to validate our proposed hypotheses, and Section 5 presents the results. Finally, this paper concludes with a discussion of the findings, implications for theory and practice and opportunities for future research in Section 6.

## 2. Literature Review and Related Works

### 2.1. Differences between Telehealth and Telemedicine

Many descriptions of telehealth and telemedicine are confusing. As these terms have become adopted in common use, they have lost their connection with the basic elements they set out to define [10]. Telehealth equipment is used as a tool for a specialist to investigate a patient with a disease condition and allows the patient to receive immediate expert advice [9].

According to the World Health Organization, telemedicine and telehealth (or the e-health system) include all healthcare providers who use information and communication technologies for the diagnosis, treatment and prevention of health services, diseases and injuries; and information and communication technologies for continuous training are the critical factors to improve the health of individuals and communities.

Telemedicine can be broadly described as the use of telecommunications technologies to provide medical services and information [18]. Although this description includes the medical-related use of a phone or fax, as well as distance learning, telemedicine is increasingly being used for remote electronic clinical deliberation [19].

Taking this information into account, we define telemedicine as paying to obtain advice from specialists in a healthcare treatment period by using videoconference, online chat (via messaging), voice call or any mode of telecommunication [18,19]. Table 1 shows more details about the terms telemedicine and telehealth.

In a 2010 study, Gudes et al. [20] reported that the field of collaborative health planning faces significant challenges created by a narrow focus on available data, a lack of a framework for organising this information and significant difficulties in accessing information and decision-making systems. Nowadays, the Internet offers many social websites with much information about healthcare, including interview videos, articles with advice from doctors and information shared by people with the same medical problems about their experiences. The World Health Organization described that if someone can quickly obtain the knowledge to solve their problem from the Internet, then they are engaging in telehealth. Telehealth includes all health issues without exception [5]. We cannot buy our health the way we can buy medicine, but health information can help us choose which healthcare path can serve us best.

Various studies have documented the effectiveness and patient satisfaction of video medical visits as opposed to face-to-face encounters [21].

### 2.2. The Role of Telehealth in Medical Tourism

Telehealth includes a wide variety of multidimensional groups of services, types of patients, specialists, methods and settings. Telehealth denotes access, as soon as possible, to medical experience and information by using information technologies.

Telehealth has two basic operational modes: deferred or asynchronous [15] and real-time or synchronous [12].

Real-time telehealth procedures may include the use of translation programmes or contacting a specialist relative (where the ‘patient’ does not need to pay for help). Real-time telehealth sessions are live connections and frequently use audio conferencing technologies.

In asynchronous [12,15] telehealth, data (e.g., digital videos, photos and reports) have been previously collected; thus, individuals can access them at any time. For example, we can check expert opinion videos and read experts’ articles, and in doing so, gather knowledge about our own health situation. If people have health problems that can easily be treated, then they can find information on how to treat them on many relevant websites. The continuous development of technology has enabled people to heal, in some cases, on their own, without the need for physicians. In this age, people who have survived any kind of illness will share their experiences with others in the virtual world. They are incredibly supportive and guide others by telling them what to do.

Telehealth is a first step to help patients ascertain their healthcare situation instantly; after that, patients can use telemedicine, which is paying money to providers to obtain suggestions. If their situation is critical, then they might decide to pursue medical tourism.

### 2.3. The Role of Telemedicine in Medical Tourism

Generally, patients do not want to break contact with their regular doctor when they choose medical tourism and go abroad. Thus, the role of telemedicine in medical tourism begins.

Telemedicine has two main steps:

(1) finding a professional specialist to discuss the problem; and
(2) paying an amount (within a certain time period).

Nord et al. evaluated the cost of a synchronised audio-video telemedicine session. They also considered patient options instead of a virtual visit, as well as the complaints of patients after the visit. All the telemedicine types included real-time audio and video. Telemedicine is a type of virtual visit, directed at the consumer, where a patient communicates with a provider through audio and video [22]. To improve telemedicine, doctors and nurses can make available real-time services from distant locations. Telemedicine can be used in almost all areas of hospital and non-hospital settings [16].

Lately, hospitals that organise medical tourism have found a great opportunity by incorporating telemedicine products into their own products. This opportunity means providing consultation remotely with patients before and after the actual medical travel. The telemedicine–medical tourism system includes tourists, tourist destinations, transit, road regions, tourist-producing regions, the tourism industry, telemedicine support systems and healthcare organisations. In this social system, well-provided call centres can serve as remote contact points for the telemedicine system [14].

Telemedicine also enables doctors to obtain information from their patients’ other medical care providers. Advances in the technology of remote monitoring can reduce the risk of hospitalisation by allowing postoperative home monitoring or can reduce the length of hospitalisation in the postoperative period [11]. As a result, telemedicine enables the development of consumer services to be offered to medical tourists. For instance, family members of patients can talk almost face-to-face via telemedicine technology with patients, surgeons or other members of the expert staff. Worried family members who could not accompany a patient may stay in communication with the patient and receive updates or briefings from the doctors or other personnel. This aspect of healthcare accelerates the healing process of treatment by ensuring that patients’ relatives can stay in touch with the patient during this critical period.

Telemedicine is the use of telecommunications technology to provide remote clinical healthcare. Telemedicine is a distant delivery of health services through telecommunications infrastructures such as counselling or health assessments. These healthcare providers evaluate, diagnose and treat patients by using common techniques such as video conferencing and smartphones, without the need to visit in person.

According to Bhattacharyya [13], a patient will have a single online environment in which the textual and the visual clinical data exchanges can take place, and in which data can be made on-demand electronically. Complete medical records, regardless of their physical location, can be available to all stakeholders as the patient receives treatment. When the patient returns to their country of residence, they may continue to receive remote follow-up care from the doctor who treated them, who can observe their healing progress.

The business process of telemedicine in medical tourism is as follows:

(1) When a patient decides to pursue medical tourism, the first business process of telemedicine as it relates to medical tourism begins. The first step is to decide how to search for a location; the patient may peruse websites of companies advertising medical tourism or that of medical tourism providers themselves. Alternatively, the patient may simply browse the Internet. In such a period of development, telecommunications technologies have become an integral part of the life and work process of patients and healthcare providers. In this way, the business role of telemedicine in medical tourism includes medical tourism companies advertising on the Internet to reach and inform people all over the world, in addition to direct communications between patients and doctors before and after the tourist visit.
(2) The second telemedicine process is the patient’s local physician actively attending the procedure, including making observations as a principal part of the recovery process.
(3) The third telemedicine process is the follow-up. Telemedicine allows virtual face-to-face communication in which the patient can contact healthcare physicians at any time, and physicians can actively participate in post-procedure and follow-up care.

### 2.4. Medical Tourism in Azerbaijan

In recent years, the rapid development of medical tourism has enabled Azerbaijan to revive its tourism sector actively. People increasingly choose Azerbaijan as a great adventure, an extraordinary vacation and a beneficial treatment location with advantages to strengthen healing. The environment, cuisine, lifestyle and nature of Azerbaijan are unique. For example, medical tourists can conclude their travel with a trip to the most popular areas of the Caucasus Mountains. The country’s mild weather and magnificent mountains, forests, waterfalls and rivers will satisfy many travellers. In 2017, Azerbaijan ranked among the top five countries in the ‘Wellness Holiday’ category of National Geographic Traveller’s awards, according to voting results over the Internet [17]. Table 2 shows a breakdown of travel to and from Azerbaijan.

As in other countries, many people in Azerbaijan want to travel to other famous medical travel destinations for medical tourism. Table 2 clearly indicates that the number of Azerbaijani citizens traveling abroad for medical care increases each year. Although Azerbaijan is not widely recognised in the medical field, the indication of foreigners traveling to Azerbaijan for health tourism is not bad either.

Health tourism in Azerbaijan improved significantly after the adoption of a state programme to develop holiday villages in 2009–2019. To strengthen the health sector, thermal tourism support unions were established in Azerbaijan, in Baku, Nakhchivan, Naftalan and other parts of the country, and modern, national medical and health centres were opened. Thermal tourism is physical therapy, rehabilitation, psychotherapy, as well as activities such as thermo-mineral water bathing, breathing and mud bathing. Azerbaijan is well-known by tourists because of its fresh mountain air, mineral waters, organic and delicious fruits and many beaches on the Caspian Sea. The Galaalti, Istisu, Duzdag and Naftalan resorts are popular worldwide [17]. Beginning in 1980, the Naftalan spas in Azerbaijan have drawn in approximately 75,000 visitors a year. Azerbaijan now has natural healthcare tourism offerings that are famous around the world. Some of them are described below.

Remedial oil treatment. Oil-rich Azerbaijan offers a rare medical product: the Naftalan crude oil, which is well-known for its curative properties. Azerbaijan has been famous for its oil for centuries, and the curative oil is transported like a precious medicine along the Silk Road. Naftalan was well-known even before the oil boom that made Baku famous worldwide in the 1800s. Naftalan oil has become famous in the West. In fact, Western-based companies began utilizing oil in Baku. Now, the industrialization and refining of Naftalan oil by Western companies have become popular as well. This oil can treat over 70 illnesses, and it is good for skin diseases.

Remedial hot water treatment. Located in Masalli, a beautiful and unique city, the Istisu resort has a name that means ‘hot water’. The location has a healing water source that cures thousands of diseases. The water contains hydrogen sulphide, sodium, chloride, calcium, magnesium hydrocarbonate and 30 milligrams of iodine per litre. The water is 60 °C. This solution can cure diseases without medication. From ancient times to the present day, local people have used this water for treatment.

Remedial salt treatment. Nakhchivan is one of the oldest regions in Azerbaijan, and home to a salt cave named Duzdag, which is located in a salt mountain. The cave, which has been used for years to treat asthma, has become a modern health centre that meets the requirements of the day. Extending 300 m into the bottom of the mountain and covered with salt rocks, the Duzdag Physiotherapy Center allows visitors to spend time in the salt cave, sometimes for many days to gain the full health benefits.

## 3. Research Model and Development of Hypotheses

In the 1950s and 1960s, John William Atkinson developed expectancy–value theory (EVT) [23] to understand individuals’ incentives for success. EVT has been developed in many different fields, including health and communication. Although models differ in meaning and implications for each area, the common opinion is that expectations, in addition to beliefs or values, influence subsequent behaviour.

In the late 1970s and early 1980s, Ajzen and Fishbein expanded EVT into theory of reasoned action [24]. Then in 1988, Ajzen presented theory of planned behaviour [25]. Planned behaviour theory and reasoned action theory [24], which are prominent theories in areas such as health communication, deal with explanatory and predictive weaknesses with EVT. Although EVT has not been used much since the early 1980s, EVT continues to be used in research in various fields, as well as in audience research.

**Figure 1:**
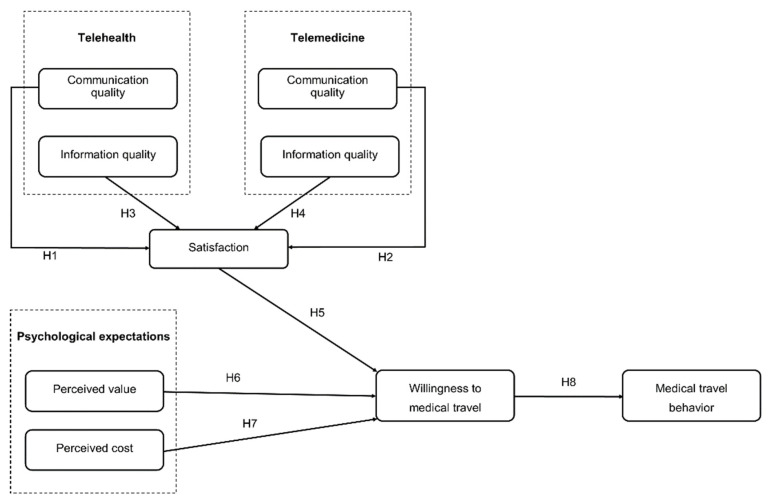
Research model.

This study was established by adapting expectation–value theory to planned behaviour theory based on various attitude theories such as reasoned action theories. The present study incorporates psychological expectation, willingness to undertake medical travel, and medical travel behaviour. These major agents affect patients’ use experiences with telehealth and telemedicine during medical tourism, and further affect patients’ willingness to continue using telehealth and telemedicine. As shown in Figure 1, this paper presents a structured model. The hypotheses are described below.

### 3.1. Relationship between Communication Quality of Telehealth and of Telemedicine and Satisfaction

Communication has important theoretical and managerial effects in the service industry. The current healthcare literature supports the critical role it plays. To achieve good communication, a common willingness and rationality should be present in successful oral and non-verbal communication exchanges [26].

Satisfaction is defined as a consumer response (cognitive or emotional), which involves comparing the actual performance of a product with the expected performance [27].

In our model, we examined the relation between the quality of communication and satisfaction, through the use of telemedicine and telehealth technology in medical tourism. Prior research on the health telecommunication system has found links between satisfaction and communication quality via the use of telemedicine and telehealth technologies [28]. Positive patient perceptions of the quality of communication should positively affect their satisfaction with the whole process. Given the use of telehealth and telemedicine in medical tourism, patients may respond favourably to the process if the communication is timely, accurate and credible. By contrast, when people perceive that communication is of low quality, they tend to be dissatisfied with the communication. We propose the following hypotheses:

**Hypothesis 1** **(H1).** *High communication quality of telehealth has a significantly positive influence on satisfaction*.

**Hypothesis 2** **(H2).** *High communication quality of telemedicine has a significantly positive influence on satisfaction*.

### 3.2. Relationship between Information Quality of Telehealth and of Telemedicine and Satisfaction

We analysed 24 articles to investigate the potential presence of contextual moderators of the relationship between the quality of information and consumer satisfaction in telehealth situations [29,30,31]. Information quality is a significant element of user satisfaction in the online situation for electronic service and retail [32] websites.

Information is a helpful service for people who are preparing to acquire a product, where ‘product’ is the basic requirement of a consumer. Therefore, although the perceived quality of online information is still important to the satisfaction of retail website consumers, other factors such as ease of ordering, transport and timely delivery may also have a role in defining satisfaction [33]. Accordingly, the quality of online information sometimes plays a more relevant role for consumers looking for information on an electronic service versus those searching for product information [34,35]. Hence, we hypothesize the following:

**Hypothesis 3** **(H3).** *High information quality of telehealth has a significantly positive influence on satisfaction*.

**Hypothesis 4** **(H4).** *High information quality of telemedicine has a significantly positive influence on satisfaction*.

### 3.3. Satisfaction and Willingness to Undertake Medical Travel

This study adopts the definition of satisfaction proposed by Anderson and Srinivasan [36]. They defined satisfaction as customer satisfaction regarding their previous experience using the communication technology of telehealth and telemedicine. According to this definition, if the actual results of the use of telemedicine and telehealth communication technology on medical tourism exceed customer expectations, then customers will be likely to be satisfied with their experience. Consequently, customers who are satisfied with their experience with telehealth and telemedicine will be motivated to continue using these applications. We described the relationship between satisfaction and willingness to undertake medical travel in our research model.

In telehealth, we can use information technology—social websites, doctor interviews—to learn about simple diseases that we can treat at home [37]. However, sometimes when illnesses are severe, the first step is to use telehealth technology to search for information from patients and medical advice from doctors recovering from the same disease and to find out which hospitals and countries treat such illnesses successfully. Essentially, this search is the first phase of a satisfaction-based relationship between telehealth and telemedicine. Therefore, satisfaction with telemedicine does not necessarily mean that patients will easily choose medical tourism.

More research is needed before going to an unknown place for treatment purposes. Therefore, after achieving satisfaction from telehealth, patients proceed with the stage of telemedicine [20].

Today, patients can easily access the contact information of the hospitals they find via telehealth. They can talk and consult with the hospital employees, sometimes even doctors, and resolve their concerns. After this satisfaction stage of using telemedicine, they can readily become willing to engage in medical tourism. Accordingly, the following hypothesis proposes that,

**Hypothesis 5** **(H5).** *User satisfaction has a significantly positive influence on willingness to undertake medical travel*.

### 3.4. Psychological Expectation and Willingness to Undertake Medical Travel

Perceived value is defined as ‘the consumer’s overall assessment of the utility of the product based on perceptions of what is received and what is given’ [38]. What is received (the benefits of a service) and what is given (the sacrifice or cost of obtaining and using the service) determine the net value of the customer service based on customer assessment [39].

In the context of services provided electronically, the analysis of models of technology acceptance shows the positive impact of perceived value on users’ behaviour. Specifically, willingness to engage in medical tourism will increase when people perceive that medical tourism services are a valuable and useful means to obtain treatment, and when they perceive that health services provide a convenient and efficient public service. Thus, the following hypothesis is proposed:

**Hypothesis 6** **(H6).** *According to the psychological expectations of the consumer, the perceived value to medical services has a significantly positive influence on willingness to undertake medical travel*.

Many researchers have examined the concept of willingness to pay for travel information systems [40]. Perceived cost predicts consumer repurchase loyalty, word-of-mouth advertising and intention. Park et al. calculated intended consumer behaviour in terms of future purchase behaviour and suggestion behaviour [41]. A favourable experience, resulting in good value for the cost, creates a positive condition and positive behavioural intention [42]. The tourism and travel literature states that consumers’ recurrent visits to a destination, and later purchase of services, rely on their satisfaction, the perceived cost and the experiences they gained during their first visit [43]. As individuals traveling for traditional reasons and those traveling for low-cost health services have dissimilar expectations, their perceptions of the value for the money spent will differ; this difference is expected to produce different behavioural intents and user satisfaction levels. Therefore, this study indicates that willingness to undertake medical travel is the perceived positive value for cost and hypothesizes that:

**Hypothesis 7** **(H7).** *International medical tourists’ repeat visits positively influence their willingness to pay more*.

### 3.5. Willingness to Undertake Medical Travel and Medical Travel Behaviour

Most travel behaviour patterns are established with the impression that adopting a specific behaviour results from a rational process that follows a logical and targeted sequence. This process takes into account behaviour options, evaluates the results of each and decides whether to act or not. The resolution of this process is commonly known as behavioural intention. This method is probably the best example of Ajzen and Fishbein’s [25] reasoned action theory [24], and updates Ajzen’s theory of planned behaviour; it is also characteristic of a number of social psychological speculations. A fundamental principle of these logical approaches is that given that all behaviours involve pre-planning, willingness to do a particular next action is based on the intention of an individual to participate in this action. These methods are successful in estimating behaviour. This success is not surprising, considering that behaviours are logical and limitless intentional behaviours. They also fit well to a rational framework with a goal-oriented approach. As Ajzen suggested [25], ‘Frankly speaking, each expected behaviour is an objective whose realization is subject to some uncertainty degree.’ Thus, we hypothesize,

**Hypothesis 8** **(H8).** *Willingness to undertake medical travel has a positive effect on medical travel behaviour and choosing a medical tourism alternative*.

## 4. Data and Methodology

### 4.1. Sampling and Data Collection

An empirical study was conducted in Azerbaijan to validate the previous research model. In this way, our questionnaire was fully supported by the Azerbaijani people. Their support helped us complete the data collection process without any problems. A multi-step iteration was used to collect data. Initially, we adopted the literature’s fundamental measurements in English (as explained in the next section), then translated the contents of the instrument through a professional Azerbaijani translation team. Later, using 40 participants, we used a pilot study to improve unclear statements, unusual expressions and the clarity of questions. We modified the questionnaire based on the data from the pilot study and the recommendations of participants. After the survey was modified, it was used to collect data from Azerbaijani residents. We completed our research using a two-stage survey. The first stage collected information except data on behaviour. After three months, we conducted the second-stage survey and collected data on behaviour. We matched the data from the two stages using the last four digits of phone numbers. This process is a novel aspect of our research. The data collection process lasted almost four months, from May 2019 to September 2019.

Small gifts were given to participants for their participation. Each gift had a value of approximately RMB 50.00 (US $7.00). The questionnaire was distributed randomly to participants. A total of 617 questionnaires were distributed, and 500 questionnaires were answered, with an 81% response rate. Table 3 shows the demographic characteristics of participants of this study.

### 4.2. Measures

Suitable variables were carefully chosen from previous studies and altered to align with the healthcare field. Nine variables were measured in this study: the communication quality of telemedicine, communication quality of telehealth, information quality of telemedicine, information quality of telehealth, satisfaction, willingness to undertake medical travel, perceived value, perceived cost and medical travel behaviour. The questionnaires were created using Likert scales ranging from 1 (strongly disagree) to 7 (strongly agree).

We used a five-item scale adjusted from previous studies [44] and new scales to measure telemedicine’s communication quality (CQTm). The communication quality of telehealth (CQTh) was measured on a four-item scale adjusted from former studies [44] and new scales. The information quality of telemedicine (IQTm) was measured on a five-item scale adopted from previous studies [45] and a new scale. The information quality of telehealth (IQTh) was measured on a four-item scale adjusted from previous studies [45,46]. Satisfaction (S) was measured on a four-item scale adjusted from previous studies [47,48,49]. Perceived value (PV) was measured on a three-item scale adjusted from previous studies [50]. Perceived cost (PC) was measured on a four-item scale adjusted from previous studies [51] and new scales. Willingness to undertake medical travel (MTW) was measured on a three-item scale adjusted from former studies [52,53]. Medical travel behaviour (MTB) was measured on a four-item scale adjusted from former studies [54].

## 5. Results

### 5.1. Measurement Model

Our validation evaluated the reliability of the measurements, whereas tests of the hypotheses analysed the hypotheses that we suggested. The structural equation pattern with partial least squares (PLS) was used to simultaneously evaluate making connections (the structural model) and measurement quality (the measurement model). When using an ordinary least squares estimation technique such as PLS, a set of iterative factor analyses is performed and a bootstrapping approach is used to estimate the significance of the paths (t-values) [55,56]. Previous research has shown that PLS–structural equation modelling (PLS-SEM) exceeds the problem identification model and is a strong method of analysing complex patterns using smaller samples [57]. Therefore, in this research, we used SmartPLS 3.0 to test hypotheses and assess measurement properties. Table 4 and Table 5 show the results.

### 5.2. Structural Model

**Figure 2:**
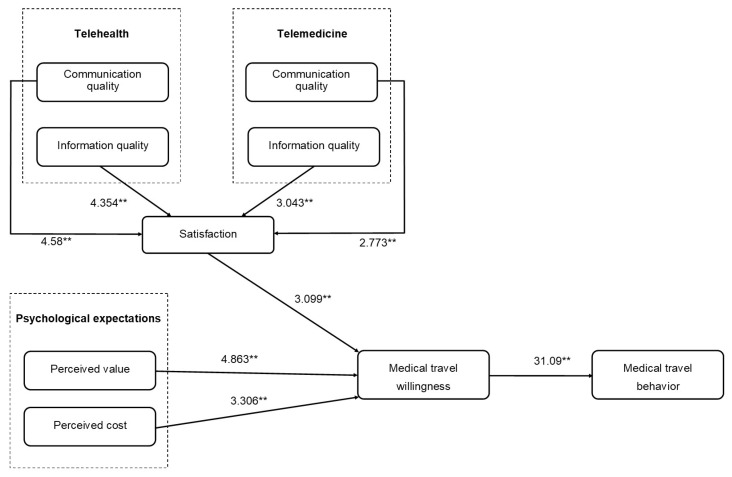
Model results. The t-values of the path coefficients are shown alongside each path. The double asterisks (**) represent *p* < 0.01.

To detect the statistical importance of the path coefficients, we used the bootstrapping method to determine the number of specimens in 5000 using 500 samples. The variables predicted in the structural model showed the direct influence of elements on other elements (Figure 2 and Table 6).

The communication quality of telehealth and communication quality of telemedicine have a significant and positive influence on satisfaction (H1 and H2) with path coefficients of 0.266 (*p* < 0.01) and 0.182 (*p* < 0.01), respectively. As predicted by H3 and H4, the information quality of telehealth and information quality of telemedicine have a significant and positive influence on satisfaction with path coefficients of 0.281 (*p* < 0.01) and 0.228 (*p* < 0.01), respectively. H5, which hypothesized a positive relationship between satisfaction and willingness to undertake medical travel, was supported (path coefficient = 0.784, *p* < 0.01). Additionally, H6, which hypothesized a positive relationship between perceived value and willingness to undertake medical travel, was supported (path coefficient = 0.186, *p* < 0.01). Perceived cost has a significant and positive influence on willingness to undertake medical travel (H7) with a path coefficient of 0.34 (*p* < 0.01). Willingness to undertake medical travel had a significant and positive influence on medical travel behaviour (H8) with a path coefficient of 0.248 (*p* < 0.01). These results show that the model fits with the data. We will discuss these findings in the subsequent section.

## 6. Discussion and Conclusions

This study focused on the effect of using telemedicine and telehealth technology on medical tourism. The empirical results support all the research hypotheses and prove the importance of telemedicine and telehealth technology use on medical tourism. Hypothesis 1 and Hypothesis 2 stated that the communication quality of telehealth and telemedicine would have a significant affirmative influence on medical tourism satisfaction [19,28]. Hypothesis 3 and Hypothesis 4 pointed out that the information quality of telehealth and telemedicine would have a significant positive effect on medical tourism satisfaction [29,34,35]. Hypothesis 5 stated that satisfaction with telehealth and telemedicine would increase the intention to choose medical tourism [36,37]. This hypothesis proved an essential satisfaction-based connection between telehealth and telemedicine. If telehealth or telemedicine is not used, then the probability of requesting medical tourism is also low. Regardless of the type of tourism, the decision to choose it readily is always based on knowledge. Similar to advertising, understanding is necessary first before the promise of attractiveness and an impressive experience at reasonable prices are considered by consumers [58]. This second part can be achieved through telemedicine with the other party/country due to the contacts that have been established. Thanks to the information that was obtained from telehealth and through our contacts with telemedicine, the sense of safety, comfort and belief can push consumers to take action.

The empirical results related to Hypothesis 6 and Hypothesis 7 show that the psycho-logical expectations of perceived value [39] and perceived cost [43] have a strong influence on willingness to undertake medical travel. These hypotheses lead to Hypothesis 8, which states that willingness to undertake medical travel positively affects medical travel behaviour [25].

From the healthcare system perspective, we studied mainly the differences between telehealth and telemedicine [11,12,13,14]. Telehealth and telemedicine are a new generation’s healthcare platforms that deliver communication between patients and hospital staff and enable people to access a variety of health information on their smartphones [12]. Telemedicine’s main features are its availability for patients and doctors, and its ability to enable comfortable communication. Telehealth gives people information about healthcare.

Our study shows that telehealth and telemedicine enhance medical travel behaviour. This result makes sense, because telehealth and telemedicine help patients determine their health situation quickly. As a result, they can seek advice from their doctor more quickly than with traditional medical practices. User satisfaction with telehealth and telemedicine helps users decide if they are willing to pursue medical travel. The psychological expectations of perceived value and perceived cost also supported the experience of user satisfaction. If patients had higher levels of satisfaction, then they were more likely to be willing to participate in medical tourism, in accordance with previous experience [36].

The results of the data analysis show a main pathway (with path coefficients over 0.25). Note that a specific individual can have a high perceived interactivity but low perceived usefulness. However, our conclusions are based on SEM-based statistical analysis. Therefore, this phenomenon with high perceived interactivity but low perceived usefulness will not influence our conclusions.

### 6.1. Theoretical and Practical Implications

The primary objective of our research was to define the mechanisms of how the information and communication qualities of the telehealth and telemedicine experiences of users affect their ultimate satisfaction with medical tourism and their willingness to pursue medical tourism. This study contributes to the existing literature in two ways and may influence future research.

Firstly, previous studies offered many definitions of telehealth and telemedicine [18,19]. However, the majority of articles stating that these two terms mean the same have limited the exact meaning of telehealth and telemedicine [9,10]. This study attempts to expand the current line of research, focusing first on all the given definitions of telehealth and telemedicine, and the comparison (Table 1) we have made shows the differences between them.

Differences between telehealth and telemedicine and how they affect medical tourism, the focus of our study, are a new distinction; doctors and all people who need them can use these systems to receive timely information and communication. Many researchers have defined various differences between telehealth and telemedicine. However, our results and data are useful additions to future research in understanding what telehealth and telemedicine are. Our focus differed from those of other researchers; the differences in telehealth and telemedicine and their roles in the field of medical tourism were explained, making a unique contribution.

Secondly, our research model demonstrates how the satisfaction of people who use telehealth and telemedicine, due to their research through telehealth and telemedicine, will increase their willingness to go to places where they are assured of good service for their treatment. Thus, we created a holistic model to understand the contribution of telehealth and telemedicine to medical tourism.

Previous studies have found that telemedicine is an integral part of medical tourism [13]. Telehealth can develop the quality, knowledge and amount of useful healthcare service information in medical tourism applications and provide better service for patients sharing information with doctors at home and abroad. This study supplied a new outlook for researchers to study user experiences and the impact and reputation of health information technology through telehealth and telemedicine, theoretically. We investigated the positive impact of user satisfaction and their willingness to pursue medical tourism; these factors could further affect the trust and relationships between patients and doctors. Our study can help theoretically focus on the in-depth aim and effectiveness of telehealth and telemedicine in the age of online medicine.

So far, the works of Song et al. [59], Goldbach and West [60], Herrick [61] and many other researchers have mentioned telehealth and the impact of telemedicine on medical tourism. Although researchers have given peculiar definitions of these two terms in their research which may confuse them, however as a common idea, they also try to explain that telehealth and telemedicine affect medical tourism.

We adopted this situation as the main focus of our study. However, our first job was to start researching what telehealth and telemedicine exactly mean. We have created a model that proves these two distinctions and their effects on medical tourism together, the most critical factor that makes our study different from others.

Due to the development of technology over the years, people already realize the convenience of telehealth and telemedicine, and because of this, they go to medical tourism more faithfully and willingly.

For this reason, our first theoretical contribution is to help people navigate more smoothly, specifying in detail the process that people unconsciously use. Additionally, to contribute to the development of medical tourism, mainly by finding out what can be done to increase the behaviour of medical tourism respectively.

In other words, if any country wants to increase medical tourism, it should share information and provide support on platforms where people can communicate more efficiently with such institutions (creating a suitable environment for telemedicine). The other important factor is the ability to satisfy patient satisfaction (which will allow people to advise each other through telehealth).

As a result of our research, we hope that people will first fully understand the definitions of telehealth and telemedicine, and countries will be able to use such platforms more frequently to increase their medical tourism behaviour.

In practice, this research may be useful for countries, hospitals and other providers of healthcare. Telemedicine is a significant function of communication technology between patients and doctors and plays an important role in developing their relationship. Telehealth is a helpful health service for users to obtain information. Considering their favourable effects on medical travel behaviour in our empirical examinations, the perceived value, perceived cost and willingness to undertake medical travel deserve attention.

Several essential factors influence medical travel behaviour and willingness to travel, including the changing quality of telehealth and telemedicine communications and information technology. Firstly, in terms of technology features in medical tourism, hospitals need to strengthen their telemedicine systems and security management to facilitate the usage of telemedicine. Given that patients have numerous interactions with a hospital’s information administration system, risks like information attacks and leakage will adversely influence users. Thus, adequate safety measure can reduce risks in the framework and increase patient confidence and perceived trustworthiness. In addition, the telehealth information system needs to be explained well to users to improve its quality and increase satisfaction.

In terms of user experience, our research shows that communication and information quality positively impact the continuity of telehealth and telemedicine use in medical tourism and increase user satisfaction. These outcomes are somewhat similar to the results of Bhattacharyya [13] and Dávalos et al. [62] on the roles of telehealth and telemedicine in medical tourism.

### 6.2. Limitations and Future Research

This study has limitations. Firstly, the study used data from a specific country. However, the scope of the study was limited only to Azerbaijan, which wanted to collect data on the use of telehealth and telemedicine as they relate to medical travel behaviour; any researcher who wants to use our model cannot incorporate data about healthcare providers or patients. Secondly, the respondents of this study were Azerbaijani people. People in different countries may give different results. Thirdly, we used a pilot study and two-stage survey to strengthen our results. This method is also the limitation of our research. Future researchers may use one-stage survey. Finally, the sample was limited to 500 participants.

Future research directions related to the current work include the following. A different or more accurate method of sampling can be used with an increased sample size. The questionnaire in this study was limited to respondents in one country (Azerbaijan); future research could replicate the same proposed model by collecting data from other countries. We did not investigate all variables; future researchers could add variables to the model, such as the variable of perceived risk. Finally, future research could continue our study of the roles of individual satisfaction and individual telehealth, or telemedicine use on medical tourism intent. The subject of our research paper deserves additional research.

## Figures and Tables

**Table 1:** Differences between telehealth and telemedicine.

	Usage Technologies	Value	Timeline	Health Consultation Service Providers
Telemedicine	Telecommunication technologies: videoconference; voice call; online chat	Fee/Paid	Stipulated	Professional aid: specialists/doctors
Telehealth	Information technologies: social websites; interviews	Free/Unpaid	Timely	Doctor interviews: knowledge/experience shared by patients with similar diseases

**Table 2:** Breakdown of foreigners arriving to azerbaijan and azerbaijan citizens traveling abroad by purpose of trip.

Indicator	2010	2011	2012	2013	2014	2015	2016	2017	2018
**Azerbaijan Citizens Traveling Abroad**									
Leisure, recreation tourism (1000 people)	729.3	513.1	897.6	1053.9	1014.7	1045.2	1096.1	1054.9	1126.3
Medical tourism (1000 people)	43.7	92.6	116.5	169.0	169.8	140.0	192.7	189.6	288.6
Other purpose (1000 people)	19.0	144.1	132.8	130.5	174.3	159.6	190.7	190.2	691.8
**Foreigners Traveling to Azerbaijan**									
Leisure, recreation tourism (1000 people)	661.7	519.8	687.8	705.2	709.9	668.8	697.1	839.3	1042.4
Medical tourism (1000 people)	14.1	33.3	43.0	46.2	46.3	36.5	41.5	49.1	63.1
Other purpose (1000 people)	46.2	31.7	38.8	41.8	42.2	30.8	39.8	41.6	45.5

Note: Data from Azerbaijan Statistic Report: https://www.stat.gov.az/source/tourism/?lang=en (accessed on 20 May 2020).

**Table 3:** Demographics of participants.

	Category	Number (%)
Gender	Male	232 (46.4%)
	Female	268 (53.6%)
Age	<25 years	192 (38.4%)
	26–35 years	201 (40.2%)
	36–45 years	50 (10%)
	46–55 years	36 (7.2%)
	56–65 years	15 (3%)
	>66 years	6 (1.2%)
Education	Junior high school or below	17 (3.4%)
	School	75 (15%)
	High school	249 (49.8%)
	Master’s degree	117 (23.4%)
	PhD	42 (8.4%)
Telehealth or telemedicine user	Yes	335 (67%)
	No	165 (33%)
Number of times telemedicine or telehealth has been used	<5 times	65 (13%)
	5–10 times	43 (8.6%)
	>10 times	227 (45.4%)
	Never	165 (33%)
Income (RMB)	<2000 CNY	144 (28.8%)
	2000–4000 CNY	136 (27.2%)
	4000–6000 CNY	85 (17%)
	6000–8000 CNY	53 (10.6%)
	8000–10,500 CNY	27 (5.4%)
	10,500–12,500 CNY	25 (5%)
	>12,500 CNY	30 (6%)
Occupation	Student	167 (33.4%)
	Corporate employee	40 (8%)
	Civil servant	46 (9.2%)
	Institution	13 (2.6%)
	Freelancer	88 (17.6%)
	Other	146 (29.2%)
Preferred countries for medical tourism	Turkey	92 (18.4%)
	Russia	21 (4.2%)
	China	114 (22.8%)
	United States	57 (11.4%)
	Germany	110 (22%)
	France	5 (1%)
	United Kingdom	9 (1.8%)
	Canada	14 (2.8%)
	Other American countries (United States and Canada not included)	2 (0.4%)
	Other Asian countries (China not included)	15 (3%)
	Other European countries (Russia, Germany, France, United Kingdom and Turkey not included)	24 (4.8%)
	Other	37 (7.4%)

**Table 4:** Descriptive statistics of the elements measured.

Factor	Item	Loading	Cronbach’s Alpha	Composite Reliability	AVE
Communication Quality of Telehealth	CQTh1	0.868	0.901	0.931	0.77
	CQTh2	0.889			
	CQTh3	0.895			
	CQTh4	0.858			
Communication Quality of Telemedicine	CQTm1	0.867	0.866	0.909	0.714
	CQTm2	0.869			
	CQTm3	0.843			
	CQTm4	0.801			
Information Quality of Telehealth	IQTh1	0.856	0.874	0.914	0.726
	IQTh2	0.84			
	IQTh3	0.833			
	IQTh4	0.879			
Information Quality of Telemedicine	IQTm1	0.846	0.912	0.934	0.74
	IQTm2	0.872			
	IQTm3	0.869			
	IQTm4	0.863			
	IQTm5	0.851			
Medical Travel Behaviour	MTB1	0.905	0.885	0.921	0.746
	MTB2	0.9			
	MTB3	0.886			
	MTB4	0.756			
Willingness to Undertake Medical Travel	WMT1	0.885	0.825	0.896	0.741
	WMT2	0.872			
	WMT3	0.824			
Perceived Cost	PC1	0.701	0.727	0.828	0.547
	PC2	0.731			
	PC3	0.798			
	PC4	0.726			
Perceived Value	PV1	0.815	0.769	0.866	0.683
	PV2	0.823			
	PV3	0.842			
Satisfaction	S1	0.893	0.891	0.925	0.754
	S2	0.903			
	S3	0.823			
	S4	0.852			

**Table 5:** Measurement model results.

Construct	1	2	3	4	5	6	7	8	9
1. Communication Quality of Telehealth	**0.878**								
2. Communication Quality of Telemedicine	0.77	**0.845**							
3. Information Quality of Telehealth	0.843	0.781	**0.852**						
4. Information Quality of Telemedicine	0.838	0.848	0.843	**0.86**					
5. Medical Travel Behaviour	0.677	0.703	0.69	0.693	**0.864**				
6. Willingness to Undertake Medical Travel	0.583	0.62	0.595	0.616	0.784	**0.861**			
7. Perceived Cost	0.595	0.583	0.608	0.635	0.595	0.551	**0.74**		
8. Perceived Value	0.766	0.697	0.768	0.733	0.755	0.641	0.605	**0.827**	
9. Satisfaction	0.834	0.8	0.839	0.842	0.707	0.625	0.641	0.76	**0.868**

Note: The bold value in the table is the square root of the AVE of each variable.

**Table 6:** Structural parameter estimates.

Hypothesis Relationship		*t*-Value	Decision
H1	Communication Quality of Telehealth -> Satisfaction	4.58 **	Supported
H2	Communication Quality of Telemedicine -> Satisfaction	2.773 **	Supported
H3	Information Quality of Telehealth -> Satisfaction	4.354 **	Supported
H4	Information Quality of Telemedicine -> Satisfaction	3.043 **	Supported
H5	Satisfaction -> Willingness to Undertake Medical Travel	3.099 **	Supported
H6	Perceived Value -> Willingness to Undertake Medical Travel	4.863 **	Supported
H7	Perceived Cost -> Willingness to Undertake Medical Travel	3.306 **	Supported
H8	Willingness to Undertake Medical Travel -> Medical Travel Behaviour	31.09 **	Supported

Note: The double asterisks (**) represent *p* < 0.01.

## Data Availability

The data presented in this study are available on request from the corresponding author. The data are not publicly available due to the request of respondents.
